# *IDH*2 mutations in patients with normal karyotype AML predict favorable responses to daunorubicin, cytarabine and cladribine regimen

**DOI:** 10.1038/s41598-021-88120-y

**Published:** 2021-05-11

**Authors:** Marta Libura, Emilia Bialopiotrowicz, Sebastian Giebel, Agnieszka Wierzbowska, Gail J. Roboz, Beata Piatkowska-Jakubas, Marta Pawelczyk, Patryk Gorniak, Katarzyna Borg, Magdalena Wojtas, Izabella Florek, Karolina Matiakowska, Bozena Jazwiec, Iwona Solarska, Monika Noyszewska-Kania, Karolina Piechna, Magdalena Zawada, Sylwia Czekalska, Zoriana Salamanczuk, Karolina Karabin, Katarzyna Wasilewska, Monika Paluszewska, Elzbieta Urbanowska, Justyna Gajkowska-Kulik, Grazyna Semenczuk, Justyna Rybka, Tomasz Wrobel, Anna Ejduk, Dariusz Kata, Sebastian Grosicki, Tadeusz Robak, Agnieszka Pluta, Agata Kominek, Katarzyna Piwocka, Karolina Pyziak, Agnieszka Sroka-Porada, Anna Wrobel, Agnieszka Przybylowicz, Marzena Wojtaszewska, Krzysztof Lewandowski, Lidia Gil, Agnieszka Piekarska, Wanda Knopinska, Lukasz Bolkun, Krzysztof Warzocha, Kazimierz Kuliczkowski, Tomasz Sacha, Grzegorz Basak, Wieslaw Wiktor Jedrzejczak, Jerzy Holowiecki, Przemysław Juszczynski, Olga Haus

**Affiliations:** 1 Medical University of Warsaw, Warsaw, Poland; 2 Institute of Hematology and Transfusion Medicine, Warsaw, Poland; 3 Maria Sklodowska-Curie National Research Institute of Oncology, Gliwice Branch, Gliwice, Poland; 4 Medical University of Lodz, Lodz, Poland; 5 Weil Cornell Medical College, New York, NY USA; 6 New York Presbyterian Hospital, New York, NY USA; 7 Faculty of Medicine, Jagiellonian University, Cracow, Poland; 8 Faculty of Medicine, Nicolaus Copernicus University in Torun, Bydgoszcz, Poland; 9 Medical University of Wroclaw, Wroclaw, Poland; 10 Hopitaux Universitaires de Geneve, Geneve, Switzerland; 11 Nicolaus Copernicus Municipal Specialist Hospital, Torun, Poland; 12 Dr Biziel University Hospital, Bydgoszcz, Poland; 13 Medical University of Silesia, Katowice, Poland; 14 Department of Cancer Prevention, School of Public Health, Silesian Medical University, Katowice, Poland; 15 Laboratory of Cytometry, Nencki Institute of Experimental Biology, Warsaw, Poland; 16 Ryvu Therapeutics S.A., Cracow, Poland; 17 Department of Hematology and Bone Marrow Transplantation, Poznan University of Medical Sciences, Poznan, Poland; 18 Department of Hematology and Transplantology, Medical University of Gdansk, Gdansk, Poland; 19 Department of Hematology, Hospital of the Ministry of Internal Affairs and Administration with Regional Oncology Center, Olsztyn, Poland; 20 Department of Hematology, Medical University of Bialystok, Bialystok, Poland

**Keywords:** Acute myeloid leukaemia, Chemotherapy, Cancer epigenetics

## Abstract

Mutations in isocitrate dehydrogenase 1 and 2 (*IDH1/2*) genes occur in about 20% patients with acute myeloid leukemia (AML), leading to DNA hypermethylation and epigenetic deregulation. We assessed the prognostic significance of *IDH1/2* mutations (*IDH1/2*^+^) in 398 AML patients with normal karyotype (NK-AML), treated with daunorubicine + cytarabine (DA), DA + cladribine (DAC), or DA + fludarabine. *IDH2* mutation was an independent favorable prognostic factor for 4-year overall survival (OS) in total NK-AML population (p = 0.03, censoring at allotransplant). We next evaluated the effect of addition of cladribine to induction regimen on the patients’ outcome according to *IDH1/2* mutation status. In DAC group, 4-year OS was increased in *IDH2*^+^ patients, compared to *IDH-*wild type group (54% vs 33%; p = 0.0087, censoring at allotransplant), while no difference was observed for DA-treated subjects. In multivariate analysis, DAC independently improved the survival of *IDH2*^+^ patients (HR = 0.6 [0.37–0.93]; p = 0.024; censored at transplant), indicating that this group specifically benefits from cladribine-containing therapy. In AML cells with R140Q or R172K *IDH2* mutations, cladribine restrained mutations-related DNA hypermethylation. Altogether, DAC regimen produces better outcomes in *IDH2*^+^ NK-AML patients than DA, and this likely results from the hypomethylating activity of cladribine. Our observations warrant further investigations of induction protocols combining cladribine with *IDH1/2* inhibitors in *IDH2*-mutant.

## Introduction

Mutations in isocitrate dehydrogenase 1 and 2 (*IDH1/2*) genes are observed in up to 20% patients with acute myeloid leukemia (AML) and constitute an early clonal event in the evolution of this disease^1^. The most common *IDH2* mutations in AML involve arginine 140 and 172 (R140 and R172) residues, which account for over 80% of all mutated *IDH2* cases^2,3^*. IDH1* mutations occur less frequently than *IDH2* in total AML population (7.7% for *IDH1* vs 15.4% for *IDH2*) and lead to a substitution of arginine 132 for either histidine or cysteine (R132H and R132C)^4^. All mentioned pathogenic *IDH1/2* mutations occur at the conserved active site of the enzymes and endow mutant enzymes with a neomorphic activity, converting alpha-ketoglutarate (αKG) to 2-hydroxyglutarate (2HG)^4^. Accumulation of 2HG competitively inhibits the activity of αKG-dependent enzymes, including Tet methylcytosine dioxygenase 2 (TET2), engaged in DNA hydroxymethylation and histone demethylation^5^. Thus, AML cells with *IDH1/2* mutations are characterized by unique hypermethylated DNA signature, which results in blocked hematopoietic differentiation^5^.

The prognostic implications of somatic *IDH* mutations in patients with normal karyotype AML (NK-AML) remain controversial^3^. Although the co-existent aberrations, such as nucleophosmin 1 (*NPM1*) mutation and internal tandem duplication of fms-like tyrosine kinase 3 (*FLT3*-ITD), have a clear impact on clinical aggressiveness of *IDH1/2*-mutated (*IDH1/2*^+^) leukemias, even in a selected *NPM1/FLT3*-ITD NK-AML subpopulation, the prognostic impact of *IDH1/2* mutations is still very heterogenous, and the factors responsible for such prognostic discrepancies are not fully understood^2,6–10^. Since there are apparent differences in the treatment protocols between independent trials, different induction regimens might explain these conflicting results^8,11–14^.

Addition of a purine analogue cladribine to daunorubicin + cytarabine 3 + 7 protocol (DA + cladribine; DAC) is an established modification of standard AML induction regimen, supported by published clinical trials from the Polish Adult Leukemia Group (PALG)^15,16^. The activity of cladribine has been mostly attributed to increased bioactivation of AraC in leukemic blasts as well as direct inhibition of DNA synthesis^17^. Importantly, cladribine exhibits DNA hypomethylating activity due to its ability to inhibit S-adenosylhomocysteine hydrolase (SAHH) and to reduce the pool of active methyl donor S-adenosylmethionine (SAM) in leukemic cells^18–21^. Our group has demonstrated in previous PALG studies that DAC was associated with increased complete remission (CR) rate and prolonged overall survival (OS), with the most significant benefit in patients with unfavorable cytogenetics^15,16^. Recently, we have also shown that the addition of cladribine alleviated the negative effect of *FLT3-*ITD on the CR rate and OS in NK-AML patients^22^.

Given the profound metabolic and epigenetic consequences of *IDH1/2* mutations and cladribine hypomethylating properties, we hypothesized that *IDH1/2* mutant leukemic blasts may exhibit differential sensitivity to DA and DAC induction regimens. In the current study, we demonstrate that DAC induction is associated with statistically significant improvement of outcome in *IDH2*^+^ NK-AML patients when compared to standard DA regimen. Finally, we postulate that this beneficial effect toward *IDH2*^**+**^ NK-AML results from the hypomethylating activity of cladribine. With ongoing clinical studies on *IDH1/2* inhibition combined with high-intensity induction regimen for newly diagnosed AML^23–25^, our data suggest that cladribine might be a potent combination partner for multi-agent therapy of *IDH2*^**+**^ AML patients.

## Results

### Prognostic relevance of IDH1/2 mutations in the entire NK-AML population and subgroups according to NPM1/FLT3 mutational status

Of the 398 analyzed de novo NK-AML cases, 80 (20.1%) patients had missense *IDH1/2* mutations (*IDH1/2*^+^). Among the *IDH1/2*^+^ subgroup, 30 (37.5%) subjects carried *IDH1* mutations in the R132 position. Of the 50 *IDH2* + patients, 35 (43.75%) and 15 (18.75%) patients carried mutations in the R140 and R172 position, respectively. The median follow-up was 40.8 months and the median survival reached 18.8 months. The estimated 4-year survival for the whole group was 37.5% with standard error ± 3. Demographic and clinical characteristics of the patients are summarized in Table 1.

**Table 1:** Patients characteristics.

	Total no (n = 398†)	*IDH1* R132^+^ (n = 30)	*IDH2* R140^+^ (n = 35)	*IDH2* R172^+^ (n = 15)	*IDH1/2*^*−*^ (n = 315)	*P* value: *IDH1*^+^ versus *IDH1/2*^−^	*P* value: R140 *IDH2*^+^ versus *IDH1/2*^*−*^	*P* value R172 *IDH2*^+^ versus *IDH1/2*^*−*^
Median age* (years)	50	56	50	55	49	0.02	0.13	0.2^a^
< 50 years ≥ 50 years	207 (52%) 191 (48%)	9 (30%) 21 (70%)	18 (51.4%) 17 (48.6%)	3 (20%) 12 (80%)	174 (55%) 141 (45%)	0.007 –	0.7 –	0.0073 –
**NPM1**^**+/−**^**/FLT3**^**−**^**IT**D^+/−**†^								
NPM1^−^/FLT3−ITD^−^ NPM1^+^/FLT3−ITD^−^ NPM1^−^/FLT3−ITD^+^ NPM1^+^/FLT3−ITD^+^	188 (47%) 84 (21%) 41 (10%) 82 (21%)	12 (40%) 10 (33%) 2 (7%) 6 (20%)	15 (43%) 13 (37%) 0 7 (20%)	15 (100%) 0 0 0	146 (47%) 60 (19%) 38 (12%) 68 (22%)	0.47 0.07 0.3 0.5	0.66 0.01 0.01 0.5	0.0001 0.044 0.15 0.03
***CEBPA mutations***								
Double Single C-*CEBPA* Single N-*CEBPA*	24 (6.14%) 16 (4.1%) 14 (3.6%)	0 (0%) 1 (3.3%) 0 (0%)	2 (6.1%) 2 (6.1%) 2 (6.1%)	0 0 0	12 (3.8%) 13 (4.15%) 22 (7%)	0.056 – –	0.23 – –	0.093 – –
Median initial WBC (× 10^9^/L)*	64.1	77.7	23.7	2.1	73.0	0.56	0.0034	0.000003
Sex**								
F M	221 (55.5%) 177 (44.5%)	26 (52%) 24 (48%)	20 (50%) 20 (50%)	8 (53%) 7(47%)	244 (56%) 193 (44%)	0.6	0.47	0.84
FAB**††								
M0 M1 M2 M4 M5 M6	20 (5.2%) 80 (20.8%) 132 (34.4%) 98 (25.5%) 51 (13.3%) 3 (0.8%)	0 (0%) 9 (30%) 11 (37%) 7 (23%) 3 (10%) 0 (0%)	1 (2.8%) 7 (20%) 13 (37%) 10 (28.6%) 3 (8.6%) 1 (3%)	0 (0%) 7 (46.65%) 7 (46.65%) 1 (6.7%) 0 (0%) 0 (0%)	19 (6.2%) 57 (18.8%) 101 (33.2%) 80 (26.3%) 45 (14.8%) 2 (0.7%)	0.16 0.11 0.7 0.45 0.34 0.82	0.36 0.5 0.64 0.77 0.23 0.28	0.39 0.016 0.2 0.07 0.096 0.9
Induction**								
DA DAC DAF 2nd induction	191 (48%) 176 (44%) 31 (8%) 135 (36%)	11 (36.6%) 17 (56.7%) 2 (6.7%) 6 (23%)	20 (57%) 11 (31.4%) 4 (11.6%) 14 (40%)	8 (53%) 7 (47%) 0 7 (47%)	150 (47.6%) 140 (44.4%) 25 (8%) 108 (36.6%)	0.25 0.2 0.82 0.5	0.28 0.14 0.32 0.55	0.66 0.86 0.26 0.84
Time to alloHSCT* (days)	321	493	250	605	305	0.06	0.88	0.001
AlloHSCT in CR1**	126 (32%)	3 (10%)	10 (28.6%)	5 (33%)	108 (34.3%)	0.0035	0.5	0.59

alloHSCT, allogenic hematopoietic stem cell transplantation; CR1, first complete remission; F, female; M, male; FAB, French American British classification; WBC, white blood cells. * calculated using the U-Mann Whitney test; ** computed by the Fisher exact test or Chi square; † for 2 high and 1 low risk NK-AML patients missing *IDH2* mutation analysis (one received DAC and the remaining patients—DA); for 3 patients *FLT3*-ITD/*NPM1* status was not established; †† for 29 patients information on FAB status is lacking; ^a^p = 0.08 when comparison was done for R172 *IDH2*^+^ versus *IDH1/2*^*−*^ patients restricted to *NPM1*^*−*^* /FLT3*-ITD^*−*^ subgroup.

Complete remission (CR) was achieved in 300/398 (75.4%) of the study population, consistent with the previous observations^26–29^. Neither *IDH1* nor *IDH2* gene mutations impacted the probability of CR in univariate (Supplemental Table S1) and multivariate analyses (Table 2).

**Table 2:** Multivariate analysis for different genetic subgroups of total NK-AML patients. All treatment groups (DA, DAC, DAF) were included in the analysis.

End point and variables		*P* value
***Total AML***(n = 398**†**)		
**CR**	**OR (95% CI)**	
Age (continuous)	1.05 (1.03–1.072)	0.000000*
WBC (continuous)	1.008 (1.0032–1.013)	1.001*
*FLT3-*ITD^+^	1.37 (0.77–2.45)	0.27*
*NPM1*(+)	0.6 (0.35–1.4)	0.067*
*IDH2*^+^	0.84 (0.4–1.8)	0.66*
**4-year OS**	**HR (95% CI)**	
Age (continuous)	1.00 (1.00–1.00)	0.014**
WBC (continuous)	1.00393 (1.00148–1.00638)	0.0016**
*FLT3-*ITD^+^	1.69 (1.24–2.3)	0.0008**
*NPM1*(+)	0.93 (0.7–1.2)	0.62**
*IDH2*^+^	0.71 (0.47–1.09)	0.12**
**4-year OS censored at allograft**	**HR (95% CI)**	
Age (continuous)	1.024 (1.012–1.036)	0.00003**
WBC (continuous)	1.0000 (1.0000–1.000001)	0.00076**
*FLT3-*ITD^+^	1.54 (1.1–2.16)	0.011**
*NPM1*(+)	0.91 (0.67–1.26)	0.61**
*IDH2*^+^	0.6 (0.37–0.93)	0.024**
***Molecular higher risk—only NPM1***^*−*^**/FLT3-ITD**^*−*^ (n = 188)		
**CR**	**OR (95% CI)**	
Age (continuous)	1.05 (1.026–1.073)	0.0000014*
WBC (continuous)	1.006 (1.001–1.01)	0.013*
*IDH1*^+^	1.55 (0.59–4.12)	0.37*
**4-year OS**	**HR (95% CI)**	
Age (continuous)	1.026 (1.015–1.04)	0.000008**
WBC (continuous)	1.0035 (1.0014–1.0056)	0.00094**
*IDH1*^+^	1.65 (0.98–2.78)	0.058**
**4-year OS censored at allograft**	**HR (95% CI)**	
Age (continuous)	1.023 (1.01–1.04)	0.0002**
WBC (continuous)	1.005 (1.0024–1.007)	0.00012**
*IDH1*^+^	1.73 (1.02–2.9)	0.04**

CR, overall complete remission rate after all courses of inductions; OS, overall survival; allo OS, overall survival censored at allograft; RFS, cumulative incidence of relapse; SD, standard deviation; HR, hazard ratio; OR, odds ratio; CI, confidence interval. † for whole NK-AML cohort: 3 patients missing *IDH2* mutation analysis (2 of *IDH2* missing patients were HR NK-AML. 1 was LR); 3 patients missing classification according to *NPM1*/*FLT3*-ITD status; ^#^ computed by log rang test; ^##^ computed by Chi square or Fisher exact test;* computed by logistic regression analysis;

**Computed by Cox regression analysis.

**Figure 1:**
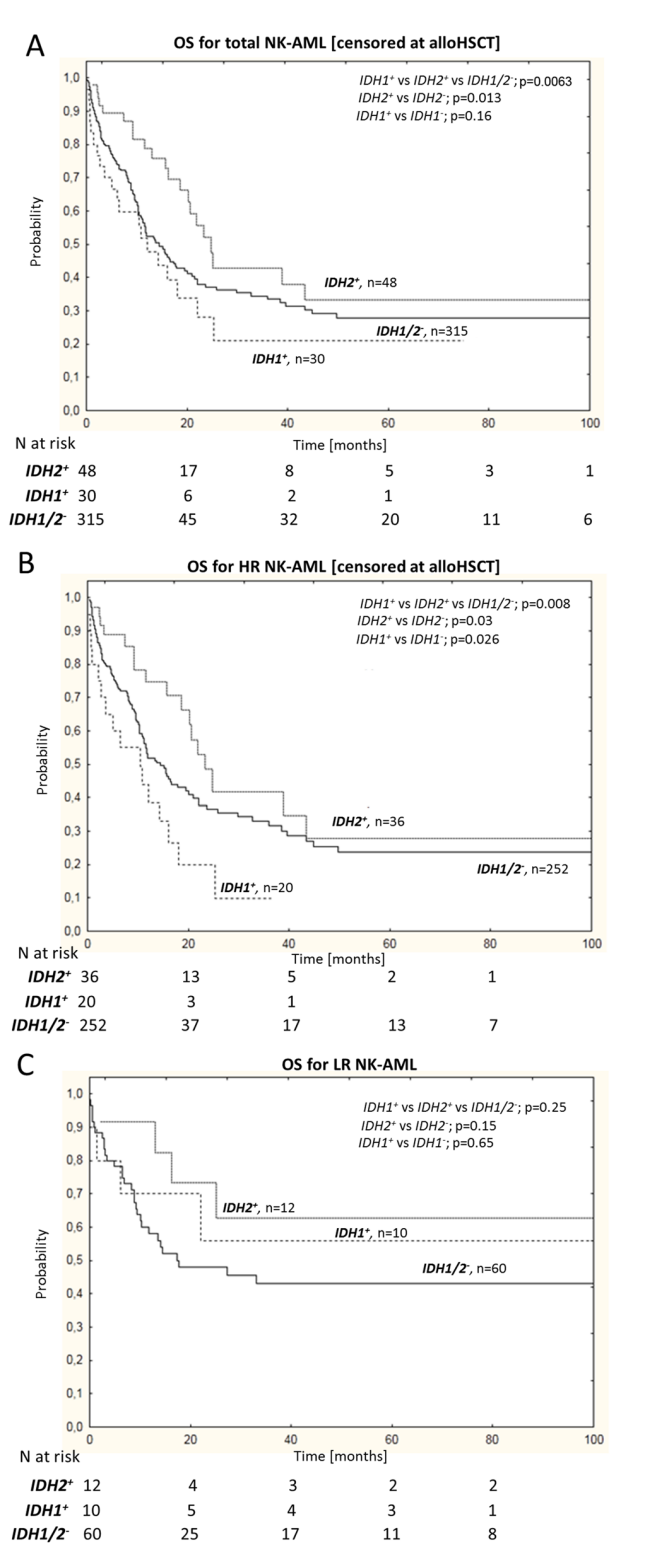
Kaplan–Meier estimates for the probability of overall survival of (**A**) total NK-AML population, as well as (**B**) high-risk and (**C**) low-risk subgroups according to *IDH1* and *IDH2* mutational status. In (**A**) and (**B**) data were censored at the time of alloHSCT. OS—overall survival, HR—high-risk AML, LR—low-risk AML; n—number of patients, p—p value.

When the entire NK-AML population was stratified according to *IDH1* mutational status, a trend towards worse overall survival (OS) was observed for *IDH1*^**+**^ subjects, although without statistical difference (Fig. 1A, Supplemental Table S1). In contrast, *IDH2*^**+**^ patients had significantly better OS in univariate (33% vs 28%; p = 0.013; Fig. 1A, Supplemental Table S1) and multivariate analyses (hazard ratio; HR:0.6 95% CI 0.37–0.93; p = 0.024; Table 2), when censored at the time of allogenic hematopoietic stem cell transplantation (alloHSCT). We next determined the influence of *NPM1/FLT3* genotype on the prognostic value of *IDH1/2* mutations. For these analyses, we compared low-risk (LR: *NPM1*^**+**^/*FLT3*-ITD^**−**^) and high-risk (HR: *FLT3*-ITD^**+**^, *NPM1*^**−**^/*FLT3*-ITD^−^) genotypes^30–34^. Mutations in *IDH1* had an adverse impact on OS in HR NK-AML patients in univariate analysis (15% vs 36% for *IDH1*^**+**^ vs *IDH1*^**−**^; p = 0.03; Fig. 1B, Supplemental Table S1). The negative effect of *IDH1* mutations was particularly significant for the *NPM1*^−^/*FLT3*-ITD^−^ genotype (OS: 15% vs 43% for *IDH1*^**+**^ vs *IDH1*^**−**^; p = 0.026; Supplemental Fig. S1A). In multivariate analysis, *IDH1* mutations had an independent prognostic impact on increased risk of death in both the HR NK-AML (p = 0.04) and *NPM1*^−^/*FLT3*-ITD^−^ (p = 0.026 with HR:2.23, 95% CI 1.1–4.54) subgroups after censoring at alloHSCT (Table 2). In contrast, *IDH2* mutations had a positive prognostic impact in both HR and LR subgroups, but only when evaluated in conjunction with *NPM1* and *FLT3* mutations (Fig. 1B,C). Neither *IDH2* R140 nor *IDH2* R172 mutations affected the survival of the *NPM1*^**−**^/*FLT3*-ITD^**−**^ subgroup (Supplemental Fig. S1C,D).

Interestingly, beyond *NPM1*^−^/*FLT3*-ITD^−^ genotype, all other *IDH2* R140 mutations from our study were always accompanied by *NPM1* mutation (with or without *FLT3*-ITD). Thus, the association between *IDH2* mutation and improved survival in the entire cohort (Fig. 1A) was driven by the favorable impact of *IDH2* mutations in the *NPM1*^**+**^/*IDH2*-R140^**+**^ genotype (Fig. 1A and Supplemental Fig. S1B). Importantly, this positive effect was not only *NPM1* mutation-dependent, but also *IDH2* mutation-specific: *NPM1*^**+**^/*IDH2*-R140^**+**^ NK-AML patients had significantly better outcomes compared to those with *NPM1*^**+**^/*IDH2*-R140^**−**^ (OS: 47% and 27% for *NPM1*^**+**^/*IDH2*-R140^+^ vs *NPM1*^**+**^/*IDH2*-R140^−^, p = 0.007) after censoring at alloHSCT (Supplemental Fig. S1B).

### Prognostic significance of IDH2 mutations in patients treated with DA versus DAC

**Figure 2:**
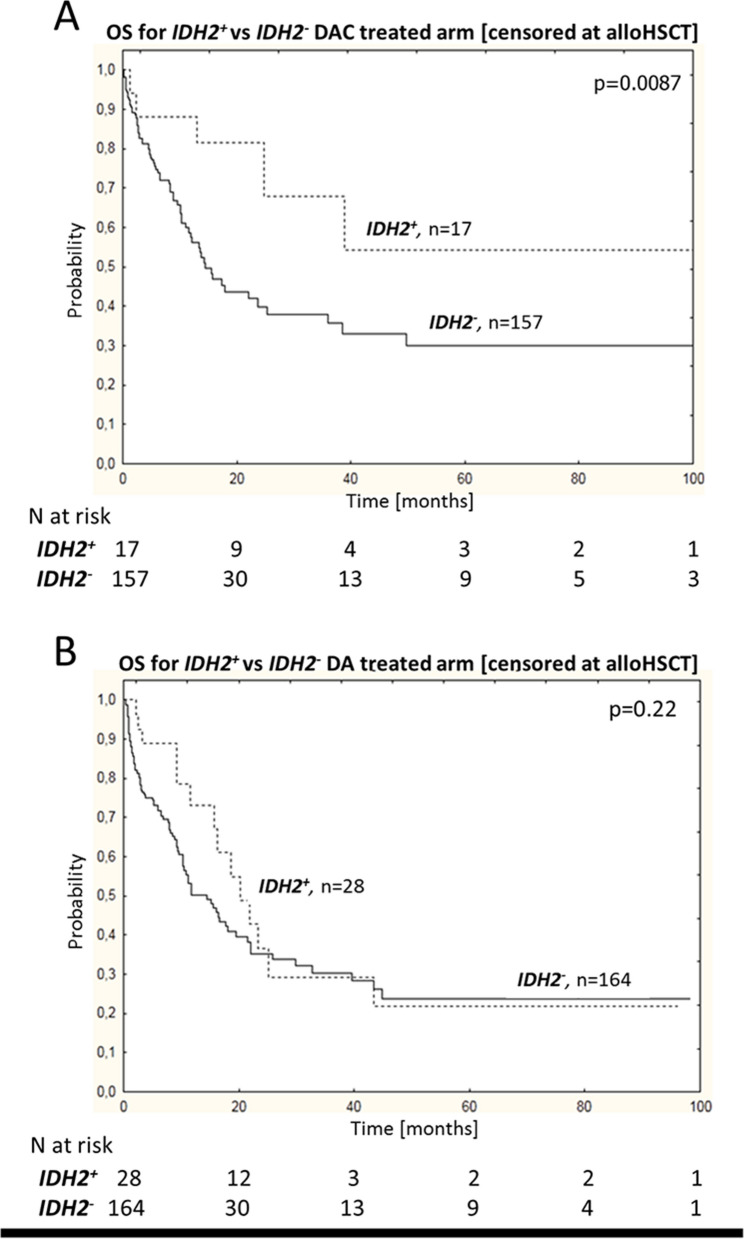
Impact of *IDH2* mutation status on survival in DAC and DA treated subgroups. (**A**) *IDH2*^+^ mutations have a positive impact on the survival of patients treated with DAC regimen. (**B**) Lack of difference in OS between *IDH2*^+^ and *IDH2*^−^ patients in DA group. OS with observations was censored at time of allo HSCT; n—number of patients, p—p value.

Interestingly, mutations in *IDH2* had a positive impact on the survival of total NK AML patients’ population treated with DAC (54% vs 33% for *IDH2*^**+**^ vs *IDH2*^**−**^, p = 0.0087) but not DA (21% vs 23% for *IDH2*^**+**^ vs *IDH2*^**−**^, p = 0.22) regimen, after censoring at alloHSCT (Fig. 2, Supplemental Fig. S2). Neither *IDH2* R140 nor *IDH2* R172 mutation had an impact on OS in DA-treated group (Supplemental Fig. S2).

### Impact of the addition of cladribine to standard DA induction on the outcome of IDH1/2^+^ NK-AML patients

**Figure 3:**
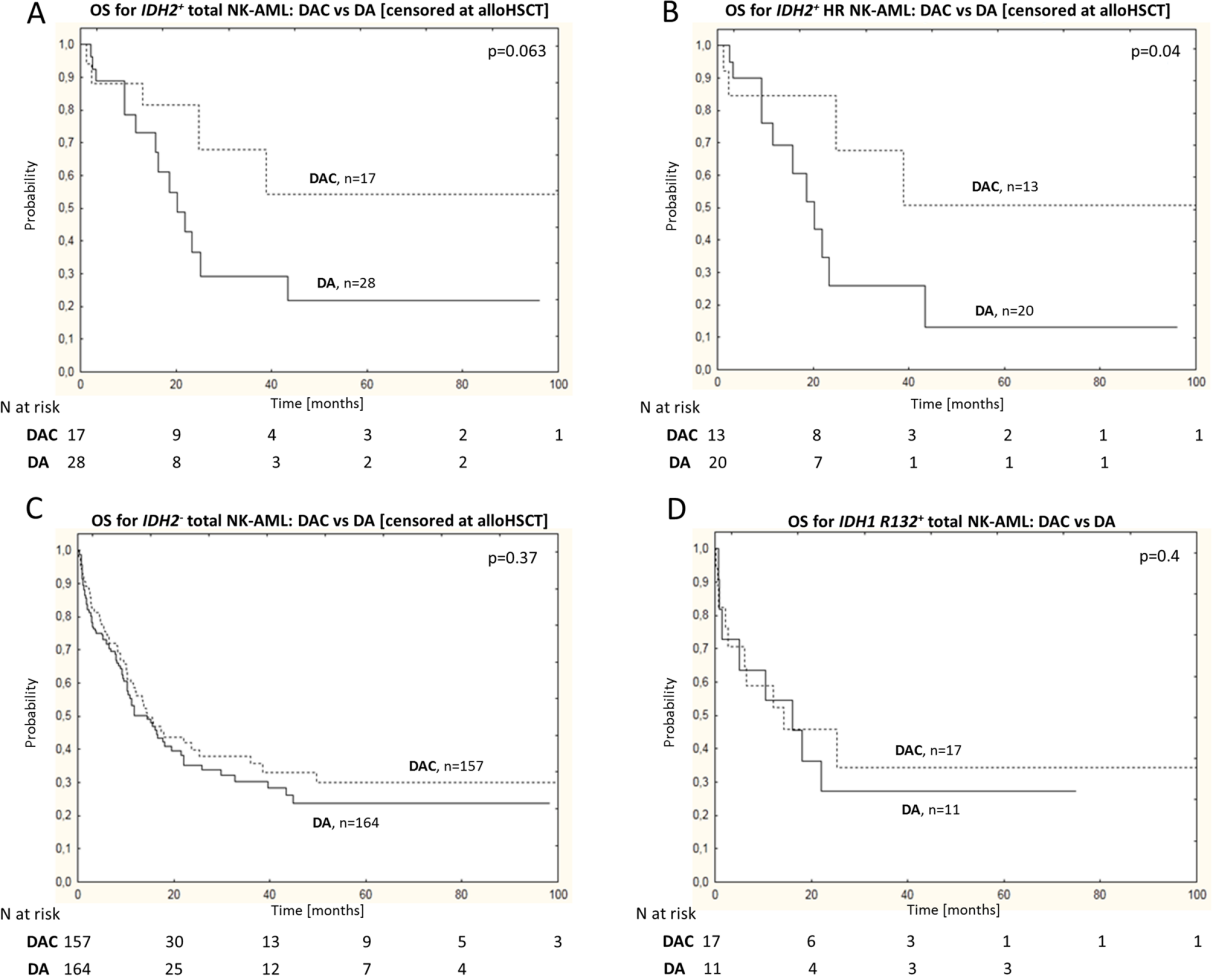
Kaplan–Meier estimates for the probability of overall survival (OS) according to induction group: DAC versus DA. Improved survival was observed in DAC treated *IDH2*^+^ NK-AML patients in total population after censoring at alloHSCT (**A**) and high risk (HR) subgroup (**B**), while no significant difference was observed for *IDH2*^−^ (**C**) and *IDH1* R132^+^ NK-AML patients (**D**). In (**A**–**C**) the observations were censored at alloHSCT; n—number of patients, p—p value.

Further we compared the clinical outcome of DAC vs DA treated *IDH1/2*^+^ patients. The DAC induction was associated with improved 4-year OS in high risk *IDH2*^+^ patients comparing to standard DA regimen after censoring for HSCT (OS: 50% vs 13% respectively; p = 0.04; Fig. 3A,B, Supplemental Table S2). Specifically, the addition of cladribine resulted in improved OS for *IDH2*^**+**^ patients in the *NPM1*^**−**^/*FLT3*-ITD^**−**^ subgroup (HR:0.3; 95% CI 0.08–0.95; p = 0.04), but not for *IDH2*^−^ or *IDH1*^+^ patients (Fig. 3C,D, Supplemental Table S2). The favorable effect of cladribine on outcome in *IDH2*^+^ subgroup was limited to younger patients (< 50 years) (Supplemental Fig. S3). However, in multivariate analysis for *IDH2*^**+**^ patients, DAC induction was independently associated with reduced risk of death when the observations were censored at alloHSCT (HR: 0.21; 95% CI 0.056–0.8; p = 0.02; Table 3).

**Table 3:** Results of multivariate analysis restricted for *IDH2*^+^ patients in different genetic subgroups of NK-AML patients.

End point and variables		*P* value
***Total NK-AML ***(n = 50)		
**CR rate after 1st induction**	**OR (95% CI)**	
Age (continuous)	0.97 (0.92–1.033)	0.38*
*CEBPA* double mut	1.06 (0.33–3.4)	0.92*
*NPM1* mut	1.9 (0.4–9.09)	0.4*
*FLT3*-ITD	1.77 (0.12–26.18)	0.66*
DAC versus DA	2.04 (0.49–8.41)	0.3*
**4-year OS**	**HR (95% CI)**	
Age (continuous)	1.04 (0.99–1.09)	0.12**
*CEBPA* double mut	1.8 (0.2–15.3)	0.57**
*NPM1* mut	0.25 (0.067–0.92)	0.038**
*FLT3*-ITD	3.2 (0.578–17.54)	0.18**
DAC versus DA	0.39 (0.14–1.1)	0.076**
**4-year OS censored at allograft**	**HR (95% CI)**	
Age (continuous)	1.03 (0.97–1.1)	0.3**
*CEBPA* double mut	1.6 (0.18–13.7)	0.66**
*NPM1* mut	0.18 (0.035–0.87)	0.03**
*FLT3*-ITD	1.26 (0.1–14.96)	0.85**
DAC versus DA	0.21 (0.056–0.8)	0.023**
***Molecular higher risk: NPM1***^*−*^**/FLT3-ITD**^*−*^ **and FLT3-ITD**^+^ (n = 37)		
**CR rate after 1st induction**	**OR (95% CI)**	
Age (continuous)	0.99 (0.92–1.05)	0.75*
*CEBPA* double mut	1.022 (0.31–3.35)	0.97*
*NPM1* mut	3.7 (0.3–46)	0.28*
DAC versus DA	2.12 (0.41–11.06)	0.34*
**4-year OS**	**HR (95% CI)**	
Age (continuous)	1.04 (0.98–1.1)	0.15*
*CEBPA* double mut	1.8 (0.2–15.3)	0.59*
*NPM1* mut	0.79 (0.22–2.87)	0.72*
DAC versus DA	0.4 (0.14–1.15)	0.09*
**4-year OS censored at allograft**	**HR (95% CI)**	
Age (continuous)	1.06 (0.98–1.15)	0.12**
*CEBPA* double mut	1.39 (0.16–12.03)	0.76**
*NPM1* mut	0.18 (0.016–2.07)	0.17**
DAC versus DA	0.15 (0.03–0.77)	0,02**

CI, confidence interval; CR, complete remission; HR, hazard ratio; OR, odds ratio; OS, overall survival; ^#^ computed by Chi square or Fisher exact test; ^##^ computed by log rank test, * computed by logistic regression analysis, ** computed by Cox regression analysis.

### Hypomethylating activity of cladribine as a possible mechanism leading to improved survival of IDH2^+^ NK-AML patients

**Figure 4:**
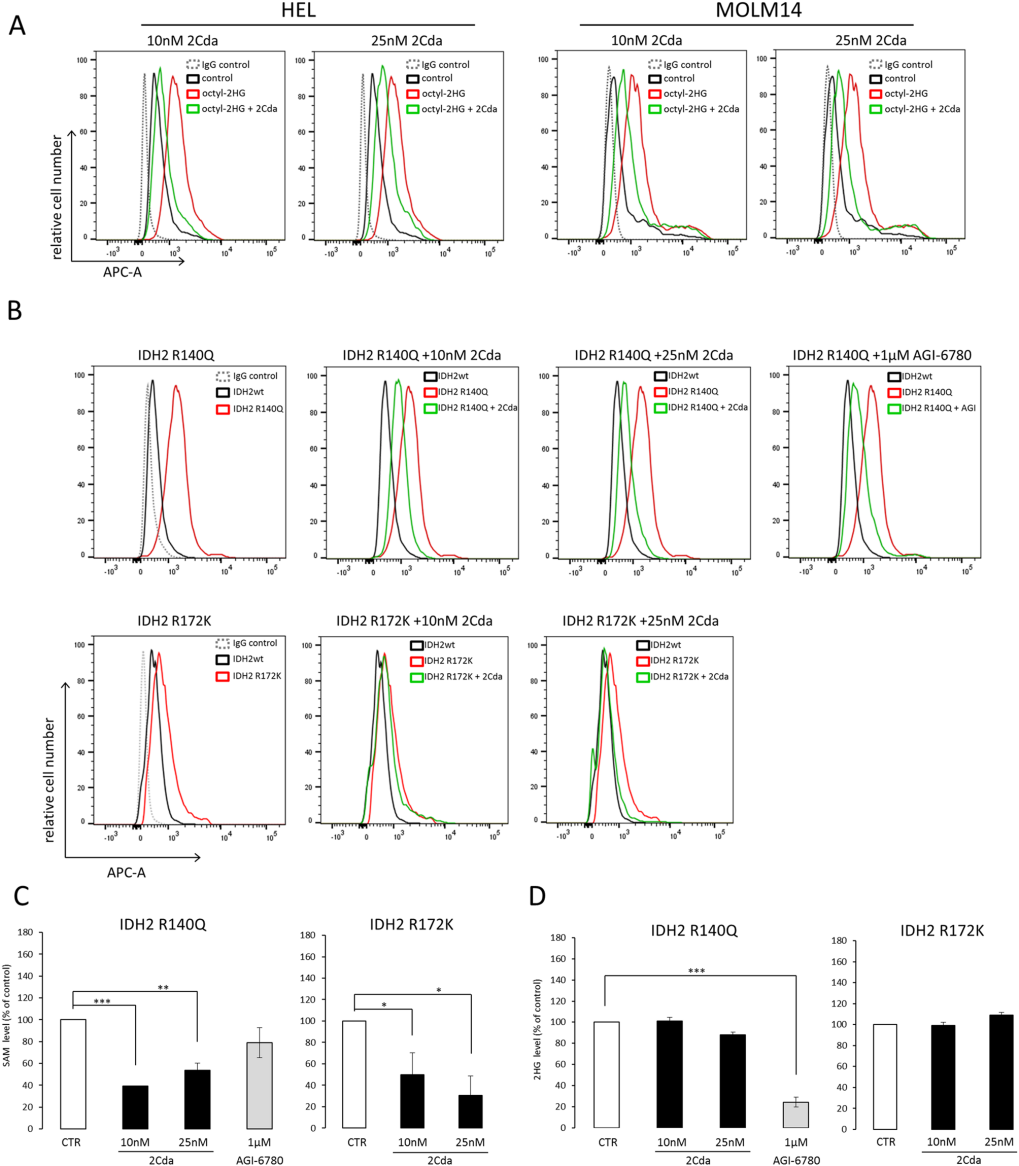
Cladribine decreases *IDH2* mutation-induced DNA hypermethylation. (**A**) Cladribine decreases DNA hypermethylation induced by incubation of HEL and MOLM14 AML cell lines with synthetic derivative of 2HG (octyl-2HG). (**B**) Cladribine restrains DNA hypermethylation induced by overexpression of *IDH2* R140Q and R172K mutants. (**C**) Cladribine reduces SAM level in *IDH2*-mutant AML cells. (**D**) In contrast to *IDH2*-mutant inhibitor AGI-6780, cladribine does not change the level of 2HG in cells overexpressing *IDH2* R140Q and *IDH2* R172K. For A and B representative histograms from 3 independent experiments were shown. Graphs in C and D show mean ± standard deviation from 3 independent experiments. *** for p < .001; ** for p < .01 and * for p < .05. Statistics was calculated with unpaired T-test.

Since our analyses indicated that cladribine was associated with improved outcomes for *IDH2*^**+**^ patients, we further investigated possible biological mechanisms underlying this phenomenon. Mutations in *IDH2* endow the enzyme with the neomorphic activity to produce 2-hydroxyglutarate (2HG), which functions as a competitive inhibitor of 2-ketoglutarate-dependent enzymes, such as TET2, a DNA-demethylating enzyme ^5^. We therefore investigated, whether cladribine could limit 2HG-dependent DNA hypermethylation in AML cells. To this end, HEL and MOLM14 cell lines were treated with synthetic cell-permeable derivative of 2HG, octyl-2HG, alone or in combination with cladribine for 24 h. For these experiments, we used low cladribine doses (10 nM and 25 nM), which were non-toxic to the cells over the 24 h treatment period (Supplemental Fig. S4). Octyl-2HG significantly increased DNA methylation, measured by 5-methylcytosine abundance, whereas simultaneous addition of cladribine suppressed DNA hypermethylation (Fig. 4A). We next tested the hypomethylating effect of cladribine in HEL cells overexpressing *IDH2* R140 and *IDH2* R172 mutants. The overproduction of 2HG in generated *IDH2* mutant cell lines was confirmed by liquid chromatography-mass spectrometry analyses (Supplemental Fig. S5). As expected, cells with *IDH2* R140 and *IDH2* R172 mutations induced DNA hypermethylation, comparing to *IDH2* wild type (IDH2wt) cells (Fig. 4B). Incubation of cells overexpressing *IDH2*-mutants with cladribine (10 nM or 25 nM, 24 h) decreased 5-methylcytosine levels comparably to the *IDH2*-R140-specific inhibitor AGI-6780 (Fig. 4B). Of note, combination of cladribine with AGI-6780 further decreased DNA methylation, as compared to the either compound used alone (Supplemental Fig. S6). Although introduction of IDH1 R132H mutation induced 2-HG production, the global DNA methylation level did not differ between the mutant and wild type cells, and remained unchanged after addition of cladribine or IDH1 R132H-targeting inhibitor (AGI-5198), (Supplemental Fig. S7). At low doses, cladribine inhibits the activity of S-adenosylhomocysteine hydrolase, a key enzyme in the biosynthesis pathway of S-adenosylmethionine (SAM), which constitutes a methyl group donor in DNA methylation reactions^18–20,35^. Therefore we determined, whether cladribine compromises DNA methylation by affecting the cellular SAM level. Consistent with our hypothesis, incubation of HEL cells overexpressing *IDH2* mutants with cladribine decreased SAM pool without influencing 2HG production, in contrast to AGI-6780, which reduced 2HG without affecting the SAM level (Fig. 4C,D).

## Discussion

The prognostic significance of *IDH1/2*-mutations in patients with NK-AML is controversial, with conflicting reports in the literature^2,8,9,12,36,37^. In the present study, we report that the impact of *IDH2* mutations on patient outcomes was related to the specific regimen used: the addition of cladribine to standard daunorubicin and cytarabine (DA) induction was independently associated with longer survival for *IDH2*^+^ patients (after censoring observations at alloHSCT). Our findings suggest that the mechanism for this beneficial effect is related to cladribine hypomethylating activity.

In our study, the *IDH2*-R140 mutation was associated with superior outcomes in the entire NK-AML, uniquely when accompanied by *NPM1* mutations, confirming the previous results^8^. Interestingly, this effect was not only *NPM1* mutation-dependent, but also *IDH2*-specific: we found the favorable effect of *NPM1* mutations only in patients with co-occurring *IDH2* mutations, suggesting synergy between the two mutations. Neither *IDH2*-R140 nor R172 impacted outcomes of patients in *NPM1*^**−**^/*FLT3*-ITD^**−**^ subgroup. These data are similar to the findings of Patel et al*.,* but different from other studies reporting a poor or uniquely favorable impact of the *IDH2* R172 mutation on prognosis^2,8,37,38^. These discrepancies may be related to study inclusion criteria, type of *IDH1/2* mutation, age, disease history as well as cytogenetic background of the analyzed population^2,8,12,14,37,39–41^. In addition, recent high-throughput sequencing studies have shown that de novo* IDH1/2*^**+**^ NK-AML frequently coexist with adverse risk-associated mutations in *DNMT3A, ASXL1, RUNX1, SRSF2, PHF6*^38,42–44^. Thus, the variable mutational spectra and co-occurring mutations in different patient cohorts may have contributed to the discrepancies in the reported prognostic impact of *IDH1/2*^**+**^ mutations between studies.

The effect of specific treatment has not been evaluated in the previous reports concerning the prognostic significance of *IDH1/2* mutations. In our study, two high-intensity induction regimens: daunorubicin + cytarabine (DA) *versus* daunorubicin + cytarabine + cladribine (DAC) were used to treat NK-AML patients^15,16^. Our analysis showed that the addition of cladribine was associated with significantly improved outcomes in *IDH2*-mutated patients. In the *NPM1*^**−**^/*FLT3*-ITD^**−**^ genotype, both *IDH2* R140 and R172 mutations showed favorable impact in the DAC-treated group, suggesting that the effect was *IDH2*-specific. Neither *IDH2* R140 nor *IDH2* R172 mutations were prognostic in the DA-treated subgroup, consistent with Patel et al.^8^. Multivariate analysis identified cladribine as an independent prognostic factor for longer survival for *IDH2*^**+**^ patients in both the entire NK-AML cohort and the *NPM1*^**−**^/*FLT3*-ITD^**−**^ subgroup. Thus, cladribine may be beneficial both in *IDH2*^**+**^ and *FLT3-*ITD^**+**^ leukemias^22^.

Intriguingly, the favorable effect of cladribine in the *IDH2*-mutated cohort was significant only when censoring for alloHSCT in most of the analyses. Therefore it is possible, that the impact of cladribine in *IDH2*^**+**^ patients is overshadowed in the setting of alloHSCT, e.g. due to improved survival of transplanted *IDH2*^***−***^ patients. Our data may also suggest that early alloHSCT in *IDH2*^**+**^ patients does not offer an advantage over chemotherapy, as has been observed for *NPM1*^**+**^ patients^26^. These possible explanations are further being investigated in an ongoing, prospective randomized clinical trial.

The mechanism of sensitivity of *IDH2*-mutant cells to cladribine is unknown. Our data show that in cells overexpressing *IDH2*-mutants, cladribine decreased SAM levels and DNA cytosine methylation, with no impact on 2HG production. Thus, in *IDH2*-mutant cells, cladribine may deplete the methyl donor pool, impair methylation reactions, and lead to decreased global DNA methylation, despite sustained production of 2HG and ongoing inhibition of 2HG-dependent enzymes, including DNA demethylases. Importantly, as concentrations similar to those used in our in vitro studies are achieved clinically using the standard doses of cladribine, corresponding levels of demethylating activity likely also occur in vivo^45^. Thus, cladribine and *IDH2* inhibitors may have different, and potentially synergistic mechanisms of DNA demethylation and our preliminary in vitro data confirmed the synergy between cladribine and *IDH2* R140Q-specific AGI-6780. Importantly, in the light of our findings, cladribine could be an interesting treatment alternative in patients with *trans* or *cis* resistance to *IDH2* inhibition^46^.

Although both *IDH2-* and *IDH1*-mutants are reported to overproduce 2HG, in our study cladribine did not improve the survival of patients with *IDH1* mutations^5,47^. Despite parallel mechanisms of transformation, *IDH1*^**+**^ and *IDH2*^**+**^ leukemias show differences in both in vitro and clinical studies. This discrepancy might be related to distinct cellular localization of IDH1 and IDH2 molecules (cytoplasmic vs mitochondrial), followed by various downstream metabolic consequences, including differential response to cytotoxic drugs^48–51^. In our in vitro IDH overexpressing model, global DNA hypermethylation was attributed only to IDH2 mutations, but not to IDH1 R132H cells. To support, although DNA hypermethylation was previously reported in both *IDH1* and *IDH2*-mutants overexpressing HEK293T cells, 5-methylcytosine level was considerably lower in *IDH1* than *IDH2*-mutants^5^. Furthermore, *IDH1*^**+**^ and *IDH2*^**+**^ leukemias differ in their mutational profiles, with high incidence of DNA (cytosine-5)-methyltransferase 3A (*DNMT3A*) mutations reported in *IDH1*^**+**^, but not *IDH2-*R140^**+**^ AML^38,43^. As *DNMT3A* mutations impact DNA methylation profile, it is very likely, that their co-segregation with *IDH1* mutations might change the response to cladribine^52^.

In summary, our data show that the addition of cladribine to standard AML induction therapy resulted in improved outcomes in patients with *IDH2* mutation. The mechanism of this synthetic effect likely involves cladribine’s demethylating activity in a molecular background of the mutation-induced DNA hypermethylation. Given the limitations of this study (retrospective nature, lack of comprehensive mutational profile at diagnosis, and relatively small *IDH1/2*^**+**^ subgroups), further investigations on cladribine as a treatment option for *IDH1/2*^**+**^ patients are warranted. Of note, a randomized, international study comparing DA *versus* DAC regimens has been already launched, with complete remission, overall survival and multimodality assessments of measurable residual disease as the study endpoints.

## Patients and methods

### Patients characteristics, material collection and molecular tests

A total of 398 de novo NK-AML patients treated in 9 PALG centers between 1999 and 2014 were either prospectively randomized to 1 of the 3 treatment groups (in the years 2000–2006): daunorubicin + cytarabine (DA; n = 18), daunorubicine + cytarabine + cladribine (DAC; n = 24), daunorubicine + cytarabine + fludarabine (DAF; n = 20), or treated outside the trial (2006–2014), according to DA (n = 173), DAC (n = 152) or DAF (n = 11) induction protocols, at the discretion of the treating physician (Table 1, Supplemental Table S3). Of note, fewer patients were included from years 2000–2006 due to limited access to molecular genetic data. Analysis of the prognostic significance of *IDH1/2* mutations was performed for the entire population (DA-, DAC- and DAF-treated; Supplemental Table S2), while the impact of cladribine on outcomes of the *IDH2*^**+**^ NK-AML population was evaluated in the DAC- *vs* DA-treated groups (Table 3 and Supplemental Table S3). All patients included in the study were eligible for intensive induction treatment with the age range from 18 to 76 years and median age of 50 years. All samples were obtained with written informed consent, in accordance with the Declaration of Helsinki. The study was approved by the local Bioethics Committees of Warsaw Medical University for all participating institutions. The mutation status of *IDH1/2* was determined as previously described^7,53,54^. Details of the material collection and molecular tests are described in Supplementary Figures and Information.

### Treatment protocols

DA consisted of daunorubicine 60 mg/m^2^ as a 5-min infusion on days 1 through 3 and a continuous infusion of cytarabine 200 mg/m^2^ on days 1 through 7. DAC additionally included cladribine (5 mg/m^2^) administered as a 3-h infusion on days 1–5^15^, while the DAF regimen consisted additionally of fludarabine 25 mg/m2 administered on days 1–5. Second courses of induction were permitted at the discretion of the treating investigator^16^. Post-remission therapy protocols were comparable in all induction groups^16^, including rates of alloHSCT (DAC, 32%; DA, 36.6%; p = 0.37). The data on *IDH1/2* mutation status and induction protocol for patients who went to transplant are given in Table 1 and Supplemental Table S1.

### Statistical analysis

The study end points were rate of complete remission (CR), median overall survival (OS), and relapse-free survival (RFS). Complete remission rate was defined according to previously published criteria^16^. Overall survival was defined as time from diagnosis to either death or last observation alive. Data analyses were performed with and without censoring the observations at the time of allogeneic hematopoietic stem cell transplantation (alloHSCT) if performed in 1st CR. Log-rank test was used to compare OS in univariate analysis. For comparison of CR rates or frequency distribution of other characteristics between subgroups, we used C*hi*-*square* or Fisher exact test (when the number of patients per subgroup was < 5). In multivariate analyses logistic regression and Cox proportional model were used to compare CR rates and OS, respectively. The statistical analyses were performed using STATISTICA 12 (StatSoft Inc. Tulsa, OK, USA).

### Chemicals, antibodies and cell culture reagents

Details on chemicals, antibodies, cell culture reagents and generation of AML HEL cells overexpressing *IDH2*-mutants are available in the Supplementary Figures and Information.

## Supplementary Information

Supplementary Information.
