# Supplementary material for: IDH2 mutations in patients with normal karyotype AML predict favorable responses to daunorubicin, cytarabine and cladribine regimen
Source: Sci Rep. 2021 May 11;11:10017. doi: 10.1038/s41598-021-88120-y (PMC8113255; doi:10.1038/s41598-021-88120-y)

**Supplementary Figures and Information**

*IDH*2 Mutations in Patients with Normal Karyotype AML Predict Favorable Responses to Daunorubicin, Cytarabine and Cladribine Regimen

Marta Libura^1^*, Emilia Bialopiotrowicz^2^, Sebastian Giebel^3^, Agnieszka Wierzbowska^4^, Gail J. Roboz^5,6^ Beata Piatkowska-Jakubas^7^, Marta Pawelczyk^1^, Patryk Gorniak^2^, Katarzyna Borg^2^, Magdalena Wojtas^2^, Izabella Florek^7^, Karolina Matiakowska^8^, Bozena Jazwiec^9^, Iwona Solarska^2^, Monika Noyszewska-Kania^2^, Karolina Piechna^2^, Magdalena Zawada^7^, Sylwia Czekalska^7^, Zoriana Salamanczuk^10^, Karolina Karabin^1^, Katarzyna Wasilewska^4^, Monika Paluszewska^1^, Elzbieta Urbanowska^1^, Justyna Gajkowska-Kulik^11^, Grazyna Semenczuk^12^, Justyna Rybka^9^, Tomasz Wrobel^9^, Anna Ejduk^2^, Dariusz Kata^13^, Sebastian Grosicki^14^, Tadeusz Robak^4^, Agnieszka Pluta^4^, Agata Kominek^15^, Katarzyna Piwocka^15^, Karolina Pyziak^16^, Agnieszka Sroka-Porada^16^, Anna Wrobel^16^, Agnieszka Przybylowicz^16^, Marzena Wojtaszewska^17^, Krzysztof Lewandowski^17^, Lidia Gil^17^, Agnieszka Piekarska^18^, Wanda Knopinska^19^, Lukasz Bolkun^20^, Krzysztof Warzocha^2^, Kazimierz Kuliczkowski^9^, Tomasz Sacha^7^, Grzegorz Basak^1^, Wieslaw Wiktor Jedrzejczak^1^, Jerzy Holowiecki^3^, Przemysław Juszczynski^2^, Olga Haus^8^

^1^ Medical University of Warsaw, Warsaw, Poland

^2^ Institute of Hematology and Transfusion Medicine, Warsaw, Poland

^3^ Maria Sklodowska-Curie National Research Institute of Oncology, Gliwice Branch, Gliwice, Poland

^4^ Medical University of Lodz, Lodz, Poland

^5^ Weil Cornell Medical College, New York, NY, US

^6^ New York Presbyterian Hospital, New York, NY, US

^7^ Faculty of Medicine Jagiellonian University, Cracow, Poland

^8^ Faculty of Medicine, Nicolaus Copernicus University in Torun, Bydgoszcz, Poland

^9^ Medical University of Wroclaw, Wroclaw, Poland

^10^ Hopitaux Universitaires de Geneve, Geneve, Switzerland

^11^ Nicolaus Copernicus Municipal Specialist Hospital, Torun, Poland

^12^ Dr Biziel University Hospital, Bydgoszcz, Poland

^13^ Medical University of Silesia, Katowice, Poland

^14^ Department of Cancer Prevention, School of Public Health, Silesian Medical University, Katowice, Poland

^15^ Laboratory of Cytometry, Nencki Institute of Experimental Biology, Warsaw, Poland

^16^ Ryvu Therapeutics S.A., Cracow, Poland

^17^ Department of Hematology and Bone Marrow Transplantation, Poznan University of Medical Sciences, Poznan, Poland

^18^ Department of Hematology and Transplantology, Medical University of Gdansk, Gdansk, Poland

^19^ Department of Hematology, Hospital of the Ministry of Internal Affairs and Administration with Regional Oncology Center, Olsztyn, Poland

^20^ Department of Hematology, Medical University of Bialystok, Bialystok, Poland

^#^ these authors jointly supervised this work

*** Correspondence:** e-mail: [marta.libura@gmail.com](mailto:marta.libura@gmail.com), tel.: 00 48 506 586 422

**Supplemental methods**

Material collection and molecular tests

Pretreatment bone marrow and/or peripheral blood specimens were enriched in mononuclear cells by Ficoll density gradient centrifugation. Genomic DNA was extracted from cryopreserved mononuclear cells by using the DNAzol reagent (Invitrogen, Karlsruhe, Germany) and total RNA was isolated using the Trizol reagent (Invitrogen, Karlsruhe, Germany), according to the manufacturer’s instructions. Patients were screened for exon 4 mutations in R132 codon of *IDH1* gene and R172 or R140 codon of *IDH2* gene by high resolution melting (HRM) analysis, followed by direct Sanger sequencing of the PCR products as previously described ^54^. The detection limit of HRM was down to 10% of mutated DNA in the background of wild-type DNA. Analyses of *NPM1* mutations and *FLT3-*ITD were performed on genomic DNA or complementary DNA with labelled primers and GeneScan method (ABI 3130 Genetic Analyser; Applied Biosystems).

Chemicals

Cladribine (2Cda) and *IDH2*-R140Q specific inhibitor AGI-6780, were from Selleck Chemicals. (2R)-octyl-α-hydroxyglutarate (octyl-2HG) was purchased from Cayman Chemicals. All chemical reagents were dissolved from lyophilized powder into culture-sterile dimethyl sulfoxide (DMSO), aliquoted according to the manufacturer’s recommendation and stored at -80°C. For each new experiment, fresh aliquots of the reagents were used. Antibody for 5-methylcytosine (ab10805) and mouse monoclonal IgG1 isotype control (ab170190) were from Abcam. APC-conjugated secondary antibody was purchased from BioLegend (cat. 405308).

Cell culture, octyl-2HG and cladribine treatment

HEL and MOLM14 cell lines were from German Collection of Microorganisms and Cell Cultures GmbH. Wild type character of the *IDH2* gene in the cell lines was confirmed by sequencing. Cell lines were grown in RPMI-1640 medium supplemented with 10% fetal bovine serum, 1% penicillin-streptomycin and 25 mM HEPES buffer. To assess changes in DNA methylation levels, HEL and MOLM14 cell lines were seeded at concentration of 0.5 x 10^6^ live cells/ml and 0.7 x 10^6^ live cells/ml, respectively and treated with 200 µM octyl-2HG alone or in combination with 10 nM and 25nM cladribine for 24 hours, then collected, and proceeded to DNA methylation analysis.

Generation of HEL cells overexpressing IDH2 mutants

The complementary DNA (cDNA) of human *IDH2*-R140Q, *IDH2*-R172K and wild type (wt) *IDH2* were designed and ordered from ATG:biosynthetics GmbH (Merzhausen, Germany) in pGH vector. The cDNA of *IDH2* was further subcloned into pMIG-IRES-EGFP vector (cat.9044, Addgene) using EcoRI and XhoI restriction enzymes (ThermoFisher Scientific) for *IDH2* variants. Obtained constructs were introduced into HEL cell line using retroviral transduction. The population positive for green fluorescent protein (GFP) expression was sorted to > 90% purity by BD FACSAria (BD Biosciences) (performed at the Laboratory of Cytometry at the Nencki Institute of Experimental Biology, Warsaw). Presence of *IDH2* mutations in generated cell lines was confirmed by sequencing.

Metabolite measurements

2-hydroxyglutarate (2HG) measurements were performed using liquid chromatography-mass spectrometry (LC-MS) on a Dionex UltiMate 3000 HPLC system coupled to a TSQ Quantiva mass spectrometer (Thermo Scientific), which was equipped with a HESI probe. Mobile Phase A consisted of 20 mM ammonium acetate, pH=6,8. Mobile Phase B was 100% acetonitrile. Each sample was injected (3 µL) onto an Acquity UHPLC HSS T3 (1.8 um particle size, 3.0x100 mm) column (Waters). The mobile phase gradient (%B) was as follows: 0 min 5%, 2 min 95%, 3 min 95%, 3.2 min 5%, 4 min 5%, all at a flow rate of 0.6 mL/min. The ionization source set was with the following parameters: ionization mode - negative, spray voltage = 3.5kV, sheath gas = 52, auxiliary gas = 16, sweep gas = 2, ion transfer tube temperature = 356°C, vaporizer temperature = 420°C. The MS data acquisition was performed by SRM mode (Single Reaction Monitoring). The precursor ion was m/z = 147.04 and products m/z = 85.11, m/z= 101.04 and m/z = 129.11. Quantitation of the data was performed with Xcalibur 4.0.27.19. SAM abundance was assessed using SAM ELISA kit (Cell Biolabs) and according to the manufacturer’s protocol.

Cell cycle and proliferation

DNA content was measured by FACS Canto II in fixed, permeabilized and propidium iodide (PI)-stained cells, as described previously ^55^. Cell cycle distribution was modelled from DNA content using FlowJo software. Proliferation was determined using MTS assay (Promega) according to the manufacturer’s protocol.

DNA meythylation (5-methylcytosine, 5mC) measurements

Cells were washed with phosphate buffered saline (PBS) and fixed with CytoFix Buffer (BD Biosciences) for 15 min in room temperature (RT). Fixed samples were centrifuged (600 g, 8 min), permeabilized (0.5% Triton X-100 in PBS, 10 min), washed with PBS, and incubated in 2 M hydrochloride acid (HCl) for DNA denaturation (RT). After 20 min samples were neutralized with 100 mM Tris-HCl pH 8.0 (15 min, RT), centrifuged, washed with PBS, and incubated with 1:100 anti-5mC antibody or 1:100 IgG isotype control (20 min, RT). After washing with PBS, cells were stained with APC-conjugated secondary antibody (1:200, 30 min, in dark), washed with PBS, and proceeded to flow cytometry analysis. Collected data were analyzed using Flow Jo software.

**Supplemental Table S1.** Univariate analysis for different genetic subgroups of NK-AML patients. All treatment groups (DA, DAC, DAF) were included in the analysis.

| **End point**  **and variables** | ***IDH1^+^***  **n=30** | ***IDH1^-^***  **n=368** | **P-value** | ***IDH2^+^***  **n=50** | | ***IDH2^-^* n=345** | **P-value** | ***IDH1/2^+^* n=80** | ***IDH1/2^-^*n=315** | | **P-value** | |
| --- | --- | --- | --- | --- | --- | --- | --- | --- | --- | --- | --- | --- |
| ***Total AML*** (n=398**†**) | | | | | | | | | | | | |
| **CR**  No of patients  (%) | 21/30  (70) | 279/368  (76) | 0.48^##^ | | 39/50  (78) | 259/345  (75) | 0.65^##^ | 60/80  (75) | 238/315 (76) | | 0.9^##^ | |
| **RFS**  No of patients  4-year rate % (SD) | 21 40 (12) | 279 50 (3) | 0.38^#^ | | 39  52 (10) | 259 49 (4) | 0.2^#^ | 60 48 (8) | 238 50 (4) | | 0.61^#^ | |
| **OS**  No of patients  4-year rate % (SD) | 30 29 (9) | 368 38 (3) | 0.18^#^ | | 50 40 (8) | 345  37 (3) | 0.09^#^ | 80 36 (6) | 315 38 (3) | | 0.62^#^ | |
| **allo OS**  No of patients  4-year rate % (SD) | 30 21 (9) | 368 29 (3) | 0.16^#^ | | 50  33 (9) | 345  28 (4) | 0.013^#^ | 80 28 (7) | 315 29 (4) | | 0.27^#^ | |
| ***Molecular Lower Risk*** (n=84**†**) ***NPM1****^+^***/*FLT3*-ITD***^-^* | | | | | | | | | | | | |
| **CR**  No of patients  (%) | 9/10  (90) | 59/74  (79.7) | 0.39^##^ | | 10/13  (77) | 57/70  (81) | 0.48^##^ | 19/23  (82.6) | | 48/60 (80) | | 0.53^##^ |
| **RFS**  No of patients  4-year rate % (SD) | 9 39 (20) | 59 63 (7) | 0.42^#^ | | 10 77 (14) | 57 58 (7) | 0.17^#^ | 19  61 (12) | | 48 61 (8) | | 0.68^#^ |
| **OS**  No of patients  4-year rate % (SD) | 10 56 (17) | 74 46 (6) | 0.65^#^ | | 13 63 (15) | n=70 45 (6) | 0.15^#^ | 23  60 (11) | | 60 43 (7) | | 0.14^#^ |
| **allo OS**  No of patients  4-year rate % (SD) | 10 46 (21) | 74 40 (7) | 0.66^#^ | | 13 49 (19) | 70 41 (8) | 0.2^#^ | 23  49 (14) | | 60 39 (8) | | 0.19^#^ |
| ***Molecular Higher Risk*** (n=311**†**) ***NPM1****^-^***/*FLT3*-ITD***^-^* **and *FLT3*-ITD***^+^* | | | | | | | | | | | | |
| **CR**  No of patients  (%) | 12/20  (60) | 217/291  (75) | 0.15^##^ | | 29/37  (78.4) | 199/272  (73) | 0.5^##^ | 41/57  (72) | 187/252 (74.6) | | 0.72^##^ | |
| **RFS**  No of patients  4-year rate % (SD) | 12 41 (15) | 217 46 (4) | 0.47^#^ | 29  42 (12) | | 199 45 (4) | 0.49^#^ | 41 40 (10) | 187 46 (4) | | 0.85^#^ | |
| **OS**  No of patients  4-year rate % (SD) | 20 15 (9) | 291 36 (3) | 0.03^#^ | 37 33 (9) | | 272  35 (3) | 0.26^#^ | 57 27 (7) | 252 36 (3) | | 0.7^#^ | |
| **allo OS**  No of patients  4-year rate % (SD) | 20 10 (8) | 291 26 (4) | 0.03^#^ | 37  28 (10) | | 272 23 (4) | 0.03^#^ | 57 19 (8) | 252 25 (4) | | 0.7^#^ | |

Abbreviations: CR - overall complete remission rate after all courses of inductions; OS - overall survival; allo OS - overall survival censored at allograft; RFS - relapse-free survival; SD - standard deviation. † for whole NK-AML cohort: 3 patients missing *IDH2* mutation analysis (2 of *IDH2* missing patients were HR NK-AML, 1 patient was LR); 3 patients missing classification according to *NPM1*/*FLT3*-ITD status; ^#^ computed by log rang test.

**Supplemental Table S2.** Results of univariate analysis for early and long-term outcome of *IDH2^+^* NK-AML patients according to the induction protocol: DA vs DAC.

| End point and variables | DAC (n=175) | DA (n=189) | P-value |
| --- | --- | --- | --- |
| *Total NK-AML* (n=364) | | | |
| CR rate after 1^st^ induction; No of patients (%) | | | |
| *IDH2^-^* | 102/157 (65%) | 87/161 (53.4%) | 0.03^#^ |
| *IDH2+* | 12/18 (67%) | 14/28 (50%) | 0.21^#^ |
| OS; No of patients; 4-year OS rate % (SD) | | | |
| *IDH2^-^* | 157; 40% (4) | 161; 36% (4) | 0.68^##^ |
| *IDH2^+^* | 17; 53% (16) | 28; 32% (9) | 0.19^##^ |
| OS censored at allograft; No of patients; 4-year OS rate % (SD) | | | |
| *IDH2^-^* | 157; 33% (6) | 161; 23% (5) | 0.37^##^ |
| *IDH2^+^* | 17; 54% (17) | 28; 21% (10) | 0.062^##^ |
| *Molecular Higher Risk NPM1^-^*/*FLT3*-ITD*^-^* and *FLT3*-ITD*^+^* (n=285) | | | |
| CR rate after 1^st^ induction; No of patients (%) | | | |
| *IDH2^-^* | 73/120 (60.8) | 67/131 (51) | 0.12^#^ |
| *IDH2^+^* | 9/13 (69) | 10/21 (47.6) | 0.19^#^ |
| OS; No of patients; 4-year OS rate % (SD) | | | |
| *IDH2^-^* | 120; 38% (5) | 131; 36% (5) | 0.81^##^ |
| *IDH2^+^* | 13; 49% (18) | 20; 23% (10) | 0.13^##^ |
| OS censored at allograft; No of patients; 4-year OS rate % (SD) | | | |
| *IDH2^-^* | 120; 28% (7) | 131; 21% (6) | 0.43^##^ |
| *IDH2^+^* | 13; 50% (19) | 20; 13% (11) | 0.04^##^ |

Abbreviations: CI - confidence interval CR - complete remission; HR - hazard ratio; OS - overall survival; SD - standard deviation. ^#^ computed by Chi square or Fisher exact test, ^##^ computed by log rank test, *^*^* computed by logistic regression analysis, *^**^* computed by Cox regression analysis.

**Supplemental Table S3.** Patients’ characteristics in different subgroups according to induction treatment.

| Induction regimen | Total No  (n=398†) | DA  (n=191†) | DAC  (n=176†) | DAF  (n=31) | P-value:  DA vs DAC |
| --- | --- | --- | --- | --- | --- |
| Median age (years)* | 50 | 44.3 | 41.4 | 31 | 0.2 |
|  | | | | | |
| NPM1+/-/FLT3-ITD+/-**† status |  |  |  |  |  |
| NPM1^-^/FLT3-ITD^-^ (%) | 188 (47) | 96 (50.5) | 78 (44.5) | 14 (49) | 0.23 |
| NPM1^+^/FLT3^-^ (%) | 84 (21) | 37 (19.5) | 42 (24) | 5 (17) | 0.3 |
| NPM1^-^/FLT3^+^ (%) | 41 (10) | 21 (11) | 15 (8.5) | 5 (17) | 0.4 |
| NPM1^+^/FLT3^+^ (%) | 82 (21) | 36 (19) | 41 (23) | 5 (17) | 0.3 |
| Median initial WBC (x10^7^)* | 64.1 | 24 | 32 | 8.3 | 0.29 |
|  |  |  |  |  |  |
| Sex** |  |  |  |  |  |
| F (%) | 221 (56) | 107 (56) | 99 (56) | 15 (48) | 0.96 |
| M (%) | 177 (44) | 84 (44) | 77 (44) | 16 (52) |  |
| alloHSCT in CR1** (%) | 126 (32) | 58 (30.3) | 60 (34) | 8 (26) | 0.44 |

Abbreviations: alloHSCT in CR1 - allogenic hematopoietic stem cell transplantation in first remission; F - female; M - male; DA - daunorubicine+cytarabine; DAC - daunorubicine+cytarabine+cladribine; DAF - daunorubicine+cytarabine+fludarabine. * computed by the U-Mann Whitney test; ** computed by the Fisher exact test or Chi square; **†** for whole NK-AML cohort: 3 patients missing *IDH2* mutation analysis (2 of *IDH2* missing patients were HR NK-AML, 1 was LR; 2 patients treated with DA, 1 treated with DAC); 3 patients missing classification according to *NPM1*/*FLT3*-ITD status

**Figure S1.** Kaplan-Meier estimates for the probability of overall survival (OS) of different *NPM1*/*FLT3*-ITD NK-AML subgroups according to *IDH1* (R132H) and *IDH2* (R140Q, R172K) mutational types. Clear adverse impact of *IDH1*-R132H mutations in HR NK-AML was even more prominent when restricting analysis to the subgroup with *NPM1*^-^/*FLT3*-ITD^-^ genotype (**A**). Among *IDH2*^+^ NK-AMLs, only *NPM1*^+^/*IDH2*-R140Q^+^ mutant patients presented favorable outcome (**B**), while both *IDH2*-R172K and *IDH2*-R140Q mutations alone, without *NPM1* and *FLT3* mutations, did not impact the survival (**C** and **D**). In A-D observations were censored at alloHSCT.


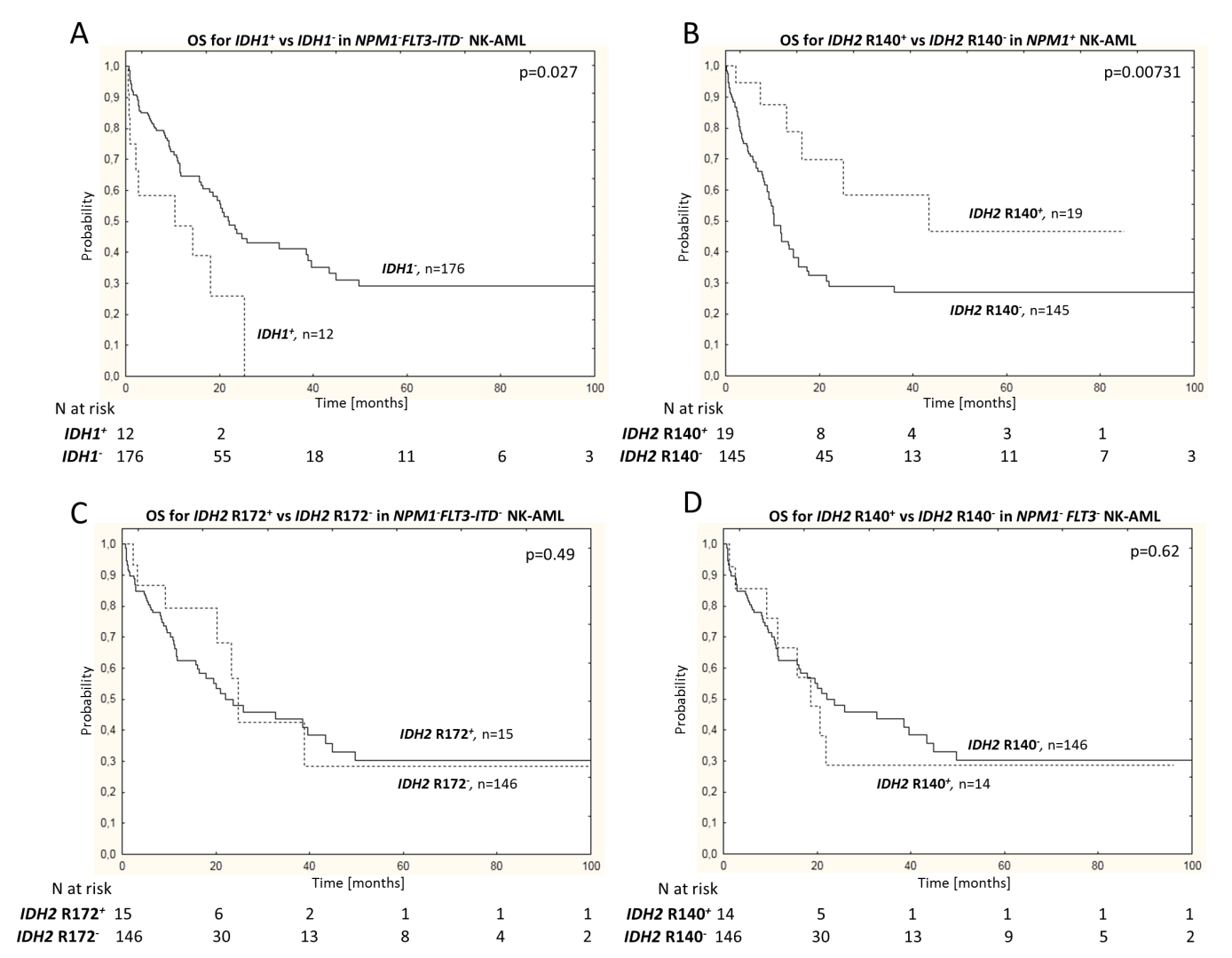


**Figure S2.** Impact of *IDH2* mutation status on survival in DAC- and DA-treated subgroups according to the mutation type. (**A**) IDH2 R140Q mutatnts presented superior overall survival (OS) independently from the induction regimen. (**B**) Positive effect of *IDH2-*R140Q mutation on survival in DAC group. (**C**) *IDH2* R140Q mutation had no impact on OS in DA-treated group. (**D**) *IDH2* R172K had no impact on OS when compared to patients without *IDH2* (*IDH2^-^*) mutations. (**E**) The impact of *IDH2* R172K mutation on OS in DAC-treated group. (**F**) *IDH2* R140Q mutation had no impact on OS in DA-treated group. In A-F figures OS was censored at the time of alloHSCT.


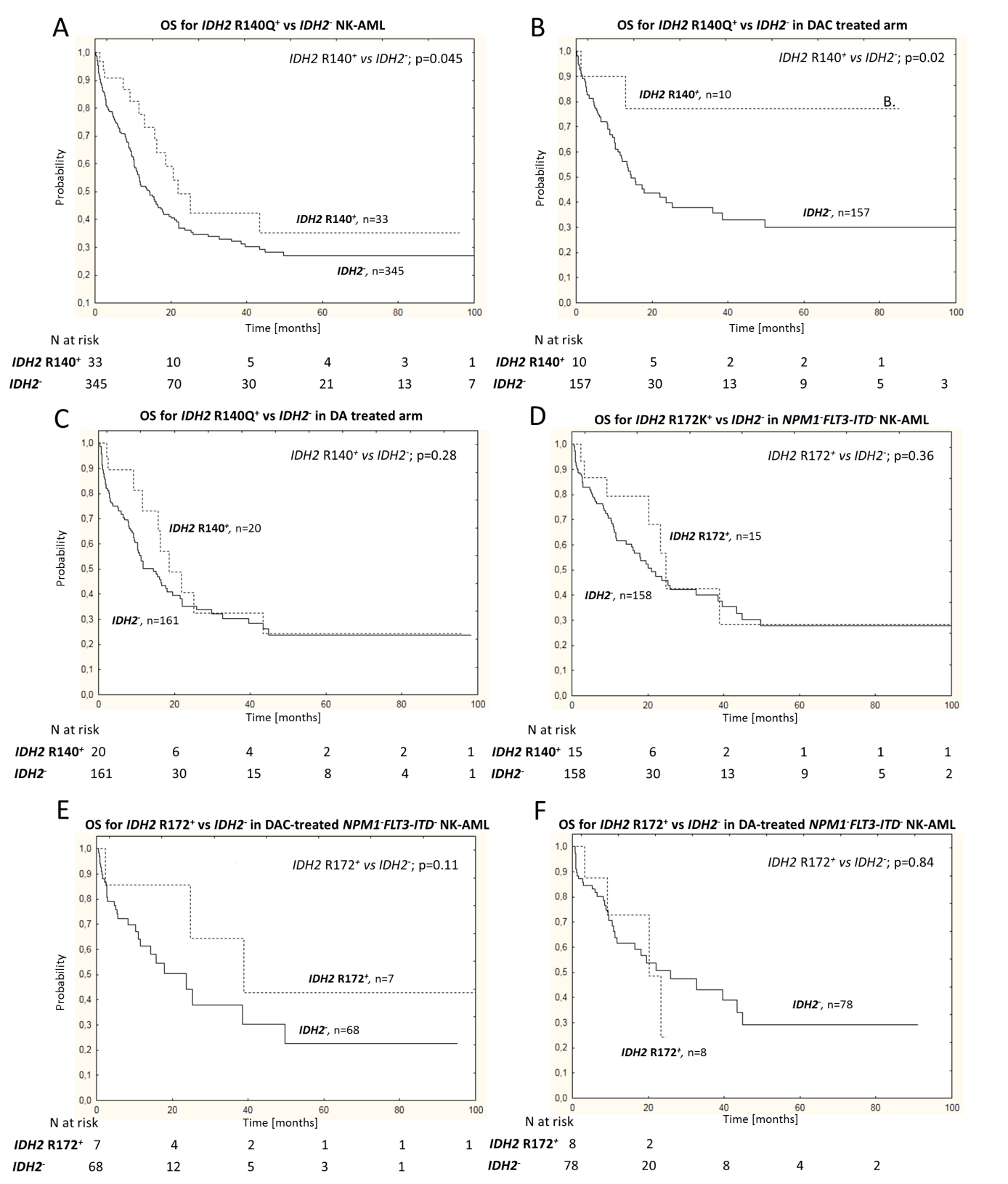


**Figure S3.** Kaplan-Meier estimates for the probability of OS according to the DAC vs DA induction in different age subgroups. Cladribine significantly improved clinical outcome in younger (<50 years old) population (**A**) when compared to the older (>50 years old) one (**B**) after censoring observations at alloHSCT.


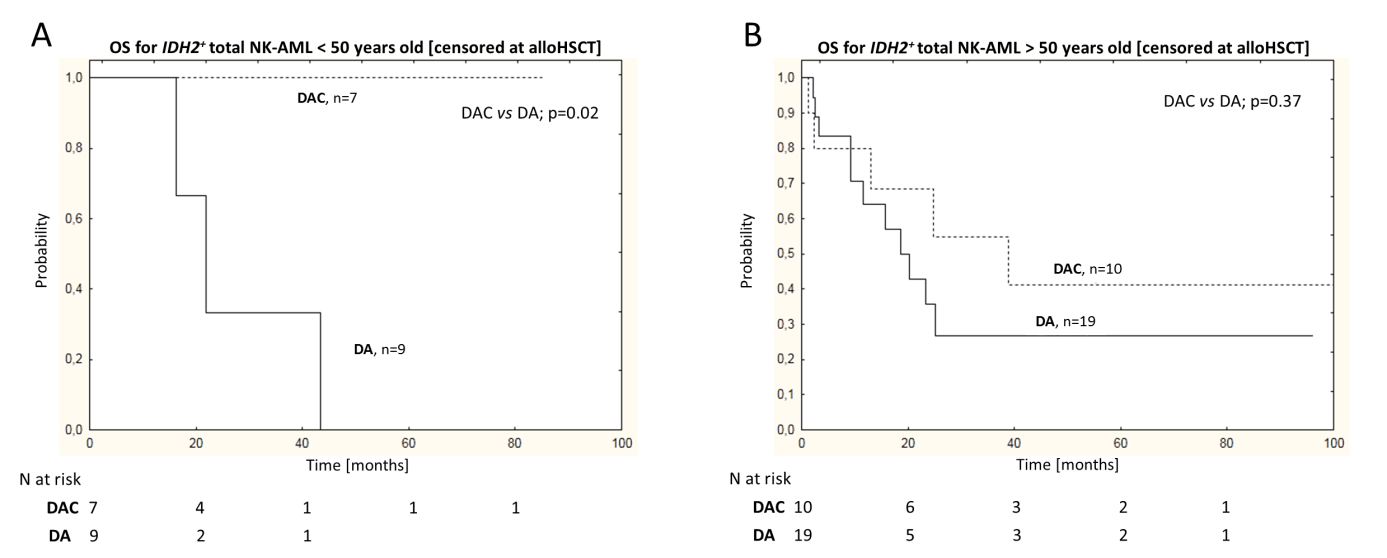


**Figure S4.** Optimization of 2Cda treatment protocol for analyses of DNA methylation. (**A**) The effect of 2Cda on AML cell lines proliferation. HEL and MOLM14 were seeded in a density of 0.5 x 10^6^ cells/ml or 0.7 x 10^6^ cells/ml respectively, and treated with increasing doses of 2Cda for 24h. Proliferation was analyzed using MTS assay. (**B**) Cell cycle analysis of HEL and MOLM14 cells treated with 200 µM octyl-2HG alone or with 2Cda (10 nM and 25 nM) simultaneously for 24h. Cell cycle was analyzed after PI staining using flow cytometry. In both experiments bars represent means ± standard deviations from 3 independent experiments.


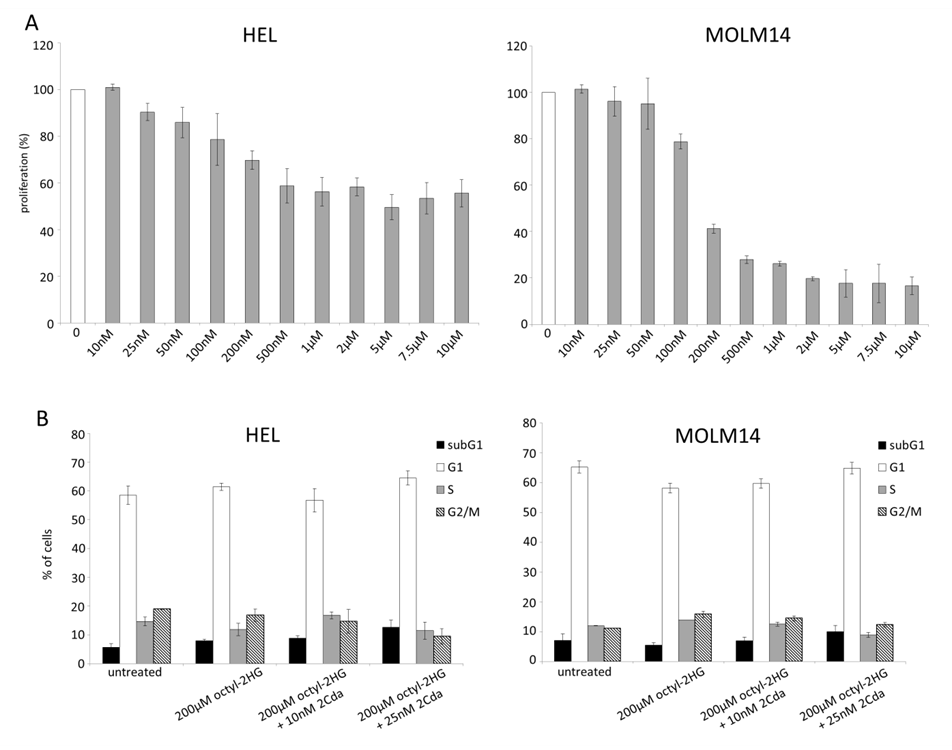


**Figure S5.** Impact of the overexpression of *IDH2* R140Q and R172K mutations in HEL cells on 2-HG levels in culture media. HEL cells were transduced with retroviral pMIG-GFP vectors bearing indicated *IDH2* mutants, sorted for GFP positive cells, and then seeded at a concentration of 0.5 x 10^6^ cells/ml for 48 hours. After that time, the supernatants were collected and proceeded to LC-MS analysis.


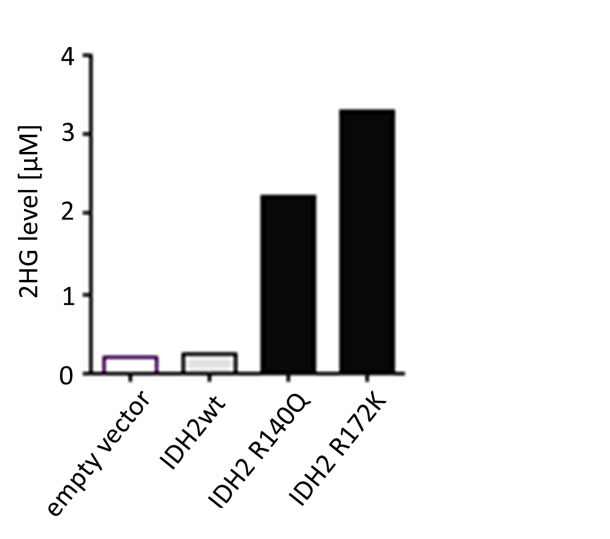


**Figure S6.** Addition of 2Cda enhances the hypomethylating effect of AGI-6780 in HEL cells overexpressing IDH2 R140Q. Cells were incubated with 10 nM 2Cda, or 1 µM AGI-6780, or both compounds for 24 hours and then assessed for 5-methylcytosine staining using flow cytometry. Representative experiment performed in triplicates was shown. Comparison between untreated and cladribine/AGI-6780 -treated cells was calculated using T-test; ** for p<.01 and * for p<.05.


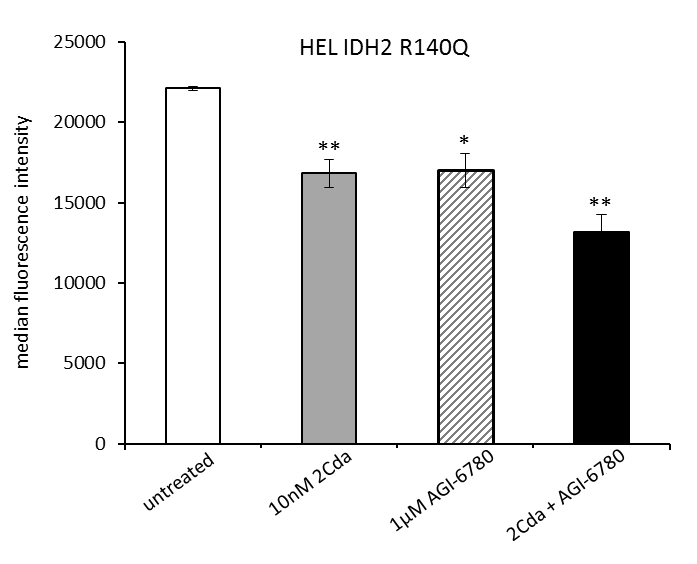


**Figure S7.** (**A**) HEL cells overexpressing IDH1 R132H mutation exhibit similar DNA methylation level comparing to IDH1 wild type (IDH1wt) overexpressing cells, which remains unchanged after incubation with cladribine (2Cda) or IDH1 R132H-targeting inhibitor (AGI-5198). Example histograms from 3 independent experiments were shown. (**B**) IDH1 R132H overexpression induces 2-HG production.


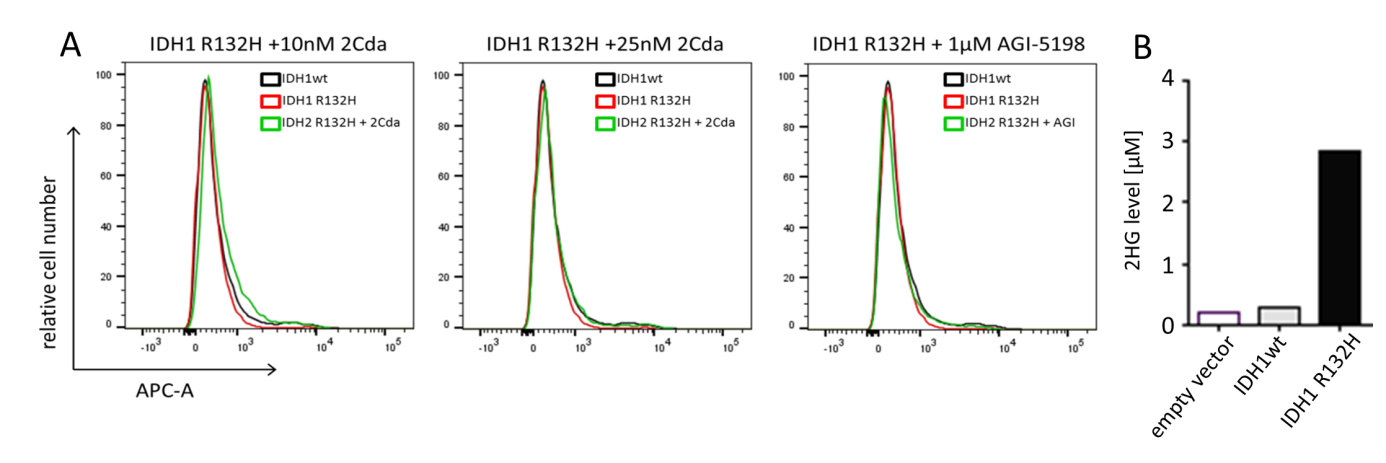

Supplement: Supplementary file 1 — Supplementary Information. [file 41598_2021_88120_MOESM1_ESM.docx]
